# Ex Vivo Lung Perfusion with β-Nicotinamide Adenine Dinucleotide (NAD^+^) Improves Ischemic Lung Function

**DOI:** 10.3390/antiox11050843

**Published:** 2022-04-26

**Authors:** Jonas Peter Ehrsam, Jin Chen, Hector Rodriguez Cetina Biefer, Isabelle Opitz, Stephan Arni, Ilhan Inci

**Affiliations:** 1 Department of Thoracic Surgery, University Hospital Zurich, 8091 Zurich, Switzerland; jonas.ehrsam@usz.ch (J.P.E.); jinchenuzh@gmail.com (J.C.); isabelle.schmitt-opitz@usz.ch (I.O.); stephan.arni@usz.ch (S.A.); 2 Department of Cardiac Surgery, City Hospital of Zurich, 8063 Zurich, Switzerland; hector.rodriguez@stadtspital.ch; 3 Deutsches Herzzentrum Berlin, 13353 Berlin, Germany; 4 Department of Cardiac Surgery, Charité Universitätsmedizin Berlin, 10117 Berlin, Germany

**Keywords:** ex vivo lung perfusion, lung transplantation, lung donation, nicotinamide adenine dinucleotide, oxidative stress, ischemia, antioxidants

## Abstract

Ischemia-reperfusion injury compromises short- and long-term outcomes after lung transplantation. The scarce existing data on NAD^+^ suggest effects on hypoxia-induced vasoconstriction, on reactive oxygen species and on tampering inflammation. We exposed rat lungs to 14 h of cold ischemic storage and perfused them in a rat ex vivo lung perfusion (EVLP) system for 4 h. A control group (*n* = 6) was compared to groups receiving 100 µM (*n* = 6) or 200 µM NAD^+^ (*n* = 6) in the preservation solution and groups receiving 200 µM (*n* = 4) or 2000 µM (*n* = 6) NAD^+^ every 30 min in the perfusate, starting at 1 h of EVLP. Compared to the control, significant effects were only achieved in the 2000 µM NAD^+^ group. During the 4 h of EVLP, we monitored higher vascular flow, lower mean pulmonary arterial pressure and increased oxygenation capacity. Tissue inflammation estimated with the myeloperoxidase assay was lower in the 2000 µM NAD^+^ group. We observed higher levels of anti-inflammatory IL-10, higher anti-inflammatory IL-6/IL-10 ratios and lower levels of pro-inflammatory IL-12 and IL-18 as well as a trend of more anti-inflammatory IFNy in the 2000 µM NAD^+^ perfusate. In the bronchoalveolar lavage, the pro-inflammatory levels of IL-1α and IL-1β were lower in the 2000 µM NAD^+^ group. NAD^+^ administered during EVLP is a promising agent with both anti-inflammatory properties and the ability to improve ischemic lung function.

## 1. Introduction

Lung transplantation is the gold-standard treatment for end-stage lung diseases. Although this procedure has undergone significant progress in terms of short and long-term survival over the past few decades, morbidity and mortality are still high [1]. One of the remaining problems for morbidity and mortality is ischemia-reperfusion injury (IRI), induced by ischemia of the donor lung during retrieval, preservation and transplantation [1,2,3,4]. Moreover chronic organ shortages have prompted surgeons to use marginal donor lungs with prolonged ischemic times [1], thus further aggravating the IRI problem.

At the beginning of ischemia, hypoxic stress causes alterations in cellular metabolism that lead to the generation of reactive oxygen species (ROS). This cascade promotes cell damage and cell death, resulting in the release of (1) endogenous pro-inflammatory mediators, (2) damage-associated molecular patterns (DAMPs) and (3) cytokines of the local innate immune cells. All of these events further increase vascular permeability and enhance leukocyte trafficking, resulting in endothelial cell dysfunction, edema, increased pulmonary vascular resistance and impaired oxygen exchange [5]. Lung IRI is regarded as the main factor for primary graft dysfunction and a major factor for the development of acute and chronic allograft rejection [4,6,7]. Currently, there are no therapeutic agents clinically designed to specifically prevent IRI, and treatment strategies are limited to supportive care.

In recent years, ex vivo lung perfusion (EVLP) has been developed as a platform for the re-evaluation and potential treatment and repair of donor lungs that are not suitable for transplantation [8].

β nicotinamide adenine dinucleotide (NAD^+^) is an essential coenzyme that regulates numerous cellular metabolic pathways. NAD^+^ is involved in the intracellular removal of ROS and re-establishes cellular homeostasis [9,10]. In addition, NAD^+^ helps counteract hypoxia-induced pulmonary vasoconstriction, which improves perfusion of the donor lung [11,12,13]. Furthermore, NAD^+^ has numerous anti-inflammatory effects on the adaptive and innate immune response [10,14,15,16]. In a murine model with ischemia selectively induced by transient ligation of a coronary artery branch, the resulting cardiac damage was significantly reduced by adding nicotinamide phosphoribosyltransferase, an enzyme that upregulates NAD^+^ production [17]. Exogenous NAD^+^ is capable of crossing the cellular membranes and gathering in the cells but especially in the mitochondria. Even nanomolar concentrations of NAD^+^ and its precursors have been found to cross membranes. However, the exact mechanism by which exogenous NAD^+^ enters the cells is not yet understood [18,19].

From these previous findings, we hypothesized that NAD^+^ might have a protective effect against ischemic injury. In this study, we tested NAD^+^ both as an additive to the organ preservation solution and during EVLP.

## 2. Materials and Methods

### 2.1. Animals

**Figure 1:**
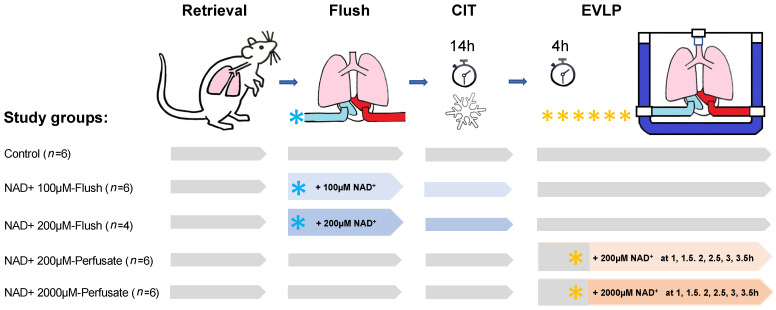
Study design. Blue and yellow stars represent the NAD^+^ dosages, the NAD^+^ application side and the NAD^+^ timing and frequencies of application. CIT, cold ischemic time; EVLP, ex vivo lung perfusion.

Male Sprague Dawley outbred rats (Janvier Labs, Le Genest Saint-Isle, France) weighing 270–300 g were maintained in a pathogen-free environment and received adequate care according to the “Guide for the Care and Use of Laboratory Animals: Eighth Edition” [20]. Figure 1 gives an overview of the study setting. The study protocol was approved by the Kanton Zurich Veterinarian committee (ZH222/18).

### 2.2. Surgical Techniques for Procurement of Lungs and EVLP Model

Rats were anaesthetized with a mixture of 2–3% (*v*/*v*) isoflurane in oxygen and underwent tracheotomy and mechanical ventilation with a rodent ventilator (Harvard Apparatus, Inc., Model Ventelite, Holliston, MA, USA). A tidal volume of 10 mL/kg was applied with a respiratory rate of 75 breaths/min at a 50:50 inspiratory/expiratory ratio, and a positive end-expiratory pressure (PEEP) of 3 cmH_2_O with a fraction of inspired oxygen (FiO_2_) of 50. Following laparotomy and sternotomy, the rats were heparinized with 300 IU intravenous heparin via the inferior vena cava. The main pulmonary artery was cannulated via incision of the right ventricle, and the left atrium cannulated via incision of the left ventricle and retrograde placement through the mitral valve. After incision of the inferior vena cava, the lung circulation was anterograde flushed with 20 mL of Perfadex plus^®^ (XVIVO Perfusion, Uppsala, Sweden) at a perfusion pressure of 20 cmH_2_O at 4 °C. Next, the trachea was clamped and the lungs inflated with a sustained airway pressure of 15 cmH_2_O. The heart lung block was explanted, weighed and immediately submerged in ice-cold Perfadex^®^ Plus solution and stored for 14 h at 4 °C. This prolonged cold ischemic time was reported to induce significant lung injuries [21].

### 2.3. EVLP Procedure and Physiological Variables

The lungs were perfused for 4 h in an isolated perfused lung system for rats and guinea pigs (IPL-2, Hugo Sachs Elektronik Harvard Apparatus, March-Hugstetten, Germany) under positive pressure ventilation and at a standard normothermic temperature of 37 °C. As a perfusate, we used 125 mL of the acellular Steen solution (Steen solution, XVIVO, Göteborg, Sweden) supplemented with 300 IU sodium heparin, antibiotic (50 mg meropenem, Labatech Pharma, Meyrin, Switzerland), and methylprednisolone (50 mg Solu-Medrol, Pfizer Inc., New York, NY, USA). The left atrium pressure was set at 2–3 cmH_2_O, and the automatized IPL-2 controller system maintained the pulmonary arterial pressure (PAP) below 15 cmH_2_O by adjusting the flow. Ventilation with the IPL-2 ventilator (VCM-P, Hugo Sachs Elektronik Harvard Apparatus, March-Hugstetten, Germany) started at a perfusate temperature of 37 °C after 20 min reperfusion time and with 30% of the targeted flow. The fixed tidal volume was at 5 mL/kg, with an inspiratory/expiratory ratio of 1/3, a rate of 30 breaths/min and with a PEEP of 3 cmH_2_O and a FiO_2_ of 0.21. Thereafter, the perfusate was continuously deoxygenated with a mixture of 8% CO_2_ and 92% N_2_ using a gas exchange membrane (D-150 hemofilter, Medsulfone, Medica S.p.A., Medolla, Italy). The 100% targeted flow was calculated as 20% of a 250 g rat with a 75 mL/min cardiac output. We started lung perfusion with 10% of the targeted flow (1.5 mL/min) for 10 min. The four following 10 min steps in mL/min were 3, 4.5, 7.5, and 12 mL/min. Then, at the 50 min time point, we switched to the maximum flow of 15 mL/min. We recorded all of the respiratory parameters with a dedicated software (PULMODYN^®^ HSE software, Hugo Sachs Elektronik Harvard Apparatus, March-Hugstetten, Germany). We monitored PAP, peak airway pressure and airway flow during EVLP. Hourly, and 5 min after switching ventilation with a FiO_2_ of 1, we recorded the dynamic lung compliance (Cdyn) and the pulmonary vascular resistance (PVR) and sampled the perfusate. At the end of the 4 h EVLP, we performed an additional stress test where the IPL-2 controller system was disabled, allowing the flow to increase over the 100% targeted flow up to the maximum PAP value set at 15 cmH_2_O [22]. At the end of this 5 min stress test, we recorded flow, PVR and Cdyn. The bronchoalveolar lavage (BAL) samples were collected after EVLP in ice-cold polypropylene tubes. For this, the right lung was clamped and a BAL of 1 mL 0.9% NaCl was taken from the left lung and centrifuged for 5 min at 4 °C at 1500 rpm. After centrifugation, only supernatants were collected. The BAL volumes recovered were estimated and were always above 90% from the starting volume and were transferred in clean polypropylene tubes that were flash-frozen in liquid nitrogen and stored at −80 °C until use. An aliquot of the right lung was flash-frozen in liquid nitrogen and stored at −80 °C until further examination. Additional tissue samples of the right lung were collected, fixed in formalin and embedded in paraffin. Tissue sections were stained with hematoxylin and eosin for microscopic assessment.

### 2.4. Methodology for NAD^+^ Application

All of the aliquoted stocks of NAD^+^ β-nicotinamide adenine dinucleotide hydrate purchased from Sigma-Aldrich (NAD^+^, N3014, St. Louis, MO, USA) were frozen at −20 °C and thawed immediately at the needed point of the experiment. For testing NAD^+^ in the preservation solution, NAD^+^ was given as an additive to 20 mL of Perfadex Plus^®^ just before anterograde flushing of the lung with the solution. The control lungs just received a flush of 20 mL of Perfadex^®^ Plus solution.

For testing NAD^+^ in the perfusate during EVLP, a volume of 1 mL Steen containing the targeted NAD^+^ concentration was injected over 5 s in the EVLP circulating perfusate at close proximity to the pulmonary artery every 30 min, meaning at 1 h, 1.5 h, 2 h, 2.5 h, 3 h and 3.5 h. During the injection period, the maximum flow of 15 mL/min was reduced for 1 min to 5 mL/min, enabling a longer NAD^+^ exposure time inside the lung capillaries.

### 2.5. Clinical Biochemistry Parameters

We used the Epoc^®^ blood analysis system (Epoc^®^ Blood Analysis System, Siemens Healthineers, Erlangen, Germany) for the pH, concentrations of calcium, potassium, glucose, sodium, and lactate, and the partial oxygen pressure. The change in pO_2_ (DpO_2_) was calculated according to the following equation: DpO_2_ = (partial pulmonary venous pO_2_ –pulmonary arterial pO_2_).

### 2.6. Thiazolyl Blue Tetrazolium Bromide Viability Assay (MTT) with a Rat Epithelial Cell Line for Non-Toxic Dosage of NAD^+^ Concentrations

As a representative of rat lung cells for this pharmacological analysis, we used the lung epithelial IL2 cell line (ATCC CCL-49) [23]. The IL2 cells were maintained in Ham’s F12 K (Kaighn’s) medium (cat#21127022, Thermo Fisher Scientific, Waltham, MA, USA ) with 10% (*v*/*v*) heat-inactivated fetal bovine serum, penicillin and streptomycin (100 ng/mL) at 37 °C in a 5% CO_2_ humidified incubator. The IL2 cells were seeded at a concentration of 0.6 × 10^5^ cells/mL in a 96-well plate. After 24 h, the cells of different wells were treated either with the Ham’s medium alone or with two-fold serial dilutions of NAD^+^ in Ham’s medium starting with the highest concentration at 500 µM. After 24 h of incubation, each well was then treated with 20 µL of tetrazolium dye MTT (3-(4,5-dethylthiazol-2-yl)-2,5-diphenyltetrazolium bromide) solution. After 4 h, the supernatants were removed, 100 µL of dimethyl sulfoxide was added to each well, and the cells were then resuspended in their wells to dissolve the precipitate. The cell viability after exposure to an increasing concentration of NAD^+^ was estimated by measuring absorbance at 570 nm using a Cytation 5 plate reader (BioTek Instruments, Inc., Winooski, VT, USA). The cell viability percentage was calculated based on the absorbance ratio between cell cultures treated with NAD^+^ and the untreated control multiplied by 100 as a representation of cell viability (percentage of control, %) from at least two separate experiments that were performed in duplicate.

### 2.7. Detection of Cytokines and Chemokines in Perfusate and BAL

For simultaneous analysis of cytokines and chemokines, a 50 µL volume either of the perfusates collected at 1 h and 4 h EVLP or of the BAL was assayed using the 27-plex Discovery assay (rat Cytokine Array/Chemokine Array 27-Plex Panel; Cat no: RD27, Eve Technologies, Alberta, ON, Canada).

### 2.8. Estimates of Myeloperoxidase Activity in Lung Tissues

Tissue lysate extracted from lung tissue (25 mg) powdered on dry ice was analyzed using a myeloperoxidase MPO activity assay (OxiSelect™ Myeloperoxidase Chlorination Activity Assay, Cell Biolabs, San Diego, CA, USA) and according to manufacturer’s instructions. The results are expressed in microUnit per milliliter of lung tissue lysate.

### 2.9. Statistical Method

Results are expressed as mean and standard deviation (SD). A nonparametric Mann–Whitney U-test or unpaired 2-tailed *t*-test were used for noncontinuous data. Data with a time component were compared using 2-way analysis of variance (ANOVA). Statistical analysis was performed with GraphPad PRISM Version 9.1.2. (GraphPad Software, Inc., La Jolla, CA, USA). Differences were considered significant at *p* < 0.05.

## 3. Results

### 3.1. In Vitro Assay to Determine the Non-Toxic Concentration of NAD^+^ for Use in the Preservation Solution

**Figure 2:**
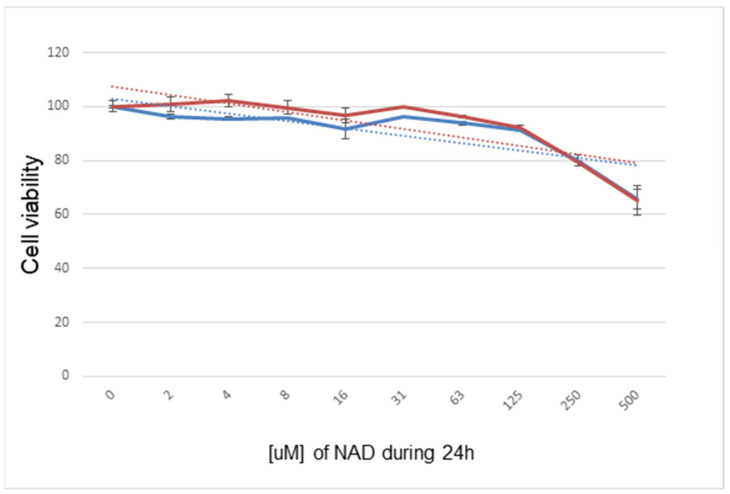
In vitro assay on functional mitochondria (MTT) in rat lung epithelial cell line IL2 over 24 h with different dosages of NAD^+^ compared to the control. Two experiments are plotted and show that NAD^+^ dosages of 250 µM and higher should be avoided.

Before the application of a defined concentration of NAD^+^ to the lung tissues in either the preservation solution or during EVLP experiments, we searched the literature for non-toxic concentrations of NAD^+^ previously used in vitro and in vivo [14,15,16]. We then designed, with the rat IL2 lung epithelial cell line as a target, the in vitro MTT viability assay based on a 24 h incubation with increasing concentrations of NAD^+^ (Figure 2). From the results of this experiment, we selected the non-toxic concentration for use in the preservation solution as being below 250 mg/kg/24 h or 250 µM, respectively.

### 3.2. Lung Physiology with NAD^+^ in the Preservation Solution during EVLP

**Figure 3:**
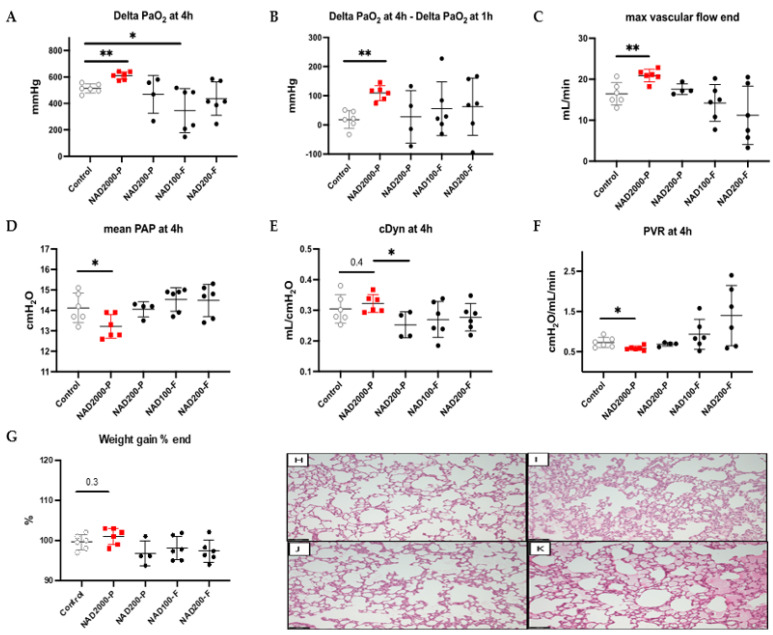
Test of 100 µM and 200 µM NAD^+^ in preservation solution vs. 200 µM and 2000 µM NAD^+^ in perfusate during EVLP with delta PaO_2_ at 4h (**A**), increase in delta PaO_2_ (delta PaO2 at 4 h–delta PaO2 at 1 h) (**B**). End of EVLP stress test for maximal vascular flow (**C**), mean PAP (**D**), cDyn (**E**) and PVR (**F**). Percentage increase in lung weight (**G**). Note * *p* ≤ 0.05; ** *p* ≤ 0.01. The stars are significant *p* values versus the control. Representative hematoxylin and eosin stained sections for assessment of lung injuries in the NAD^+^ 2000 µM perfusate (**H**), the NAD^+^ 200 µM perfusate (**I**), the NAD^+^ 200 µM flush (**J**) and the control (**K**). Sections are shown at X100 magnification at the end of the 4 h EVLP period. The scale bar indicates 100 µm length.

As additives in the preservation solution applied in the flush and present in lung vessels during 14 h of cold ischemia time, the NAD^+^ 100 µM flush and NAD^+^ 200 µM flush groups revealed, after 4 h of EVLP, heterogeneous and unbeneficial effects on physiology in comparison to the control group (Figure 3). We recorded significantly lower oxygenation capacity with 100 µM NAD^+^ flush (*p* = 0.037, Figure 3A) and a trend of worse oxygenation with 200 µM NAD^+^ flush (*p* = 0.181, Figure 3A). The two NAD^+^ flush groups had a very low initial oxygenation capacity at 1 h of EVLP, leading to a misleading comparable oxygenation increase between the first and the fourth hour of EVLP in comparison to the control group (NAD^+^ 100 µM flush *p* = 0.699; NAD^+^ 200 µM flush *p* = 0.258, respectively, Figure 3B). Maximal flow was reduced and showed a trend of being more obstructed with 200 µM NAD^+^ flush (*p* = 0.124, Figure 3C) than in the control group, whereas 100 µM NAD^+^ flush was comparable (*p* = 0.390, Figure 3C). Mean PAP and cDyn after 4 h of EVLP were comparable in both NAD^+^ flush groups in comparison to the control group (NAD^+^ 100 µM flush *p* = 0.321 and *p* = 0.285; NAD^+^ 200 µM flush *p* = 0.565 and *p* = 0.394, Figure 3D,E, respectively). At the end of the experiment, the average lung weight of the 100 µM NAD^+^ flush group was comparable to that of the control group (*p* = 0.394, Figure 3G), and in the 200 µM NAD^+^ group was not significantly lower (*p* = 0.1797, Figure 3G). In parallel, we did not observe significant differences in the microscopic assessment of lung injuries (i.e., alveolar damage or edema) at the end of the 4 h EVLP period in either the NAD^+^ 100 µM flush or the NAD^+^ 200 µM flush (Figure 3J) group, or in the control group (Figure 3K).

As neither the 100 µM nor the 200 µM NAD^+^ flush group concentrations triggered any beneficial trends, no further NAD^+^ concentrations were tested.

### 3.3. NAD+ in the Perfusate

#### 3.3.1. Lung Physiology during EVLP with NAD^+^ 200 µM Perfusate

As a next step, we tested 200 µM NAD^+^ injected bolus in the perfusate of the EVLP system in close proximity to the pulmonary artery every 30 min (at 1 h, 1.5 h, 2 h, 2.5 h, 3 h and 3.5 h of EVLP). As presented in Figure 3, we observed no difference in oxygenation at the end of the experiment (*p* = 0.943, Figure 3A), increase in oxygenation capacity (*p* = 0.914, Figure 3B), maximal flow (*p* = 0.457, Figure 3C), mean PAP (*p* = 0.999, Figure 3D), cDyn (*p* = 0.257, Figure 3E), and PVR (*p* = 0.724, Figure 3F), but a not significantly lower lung weight was recorded at the end of the experiment (*p* = 0.171, Figure 3G). The microscopic assessments were also comparable for the NAD^+^ 200 µM-perfusate (Figure 3I) and the control group (Figure 3K). From the results of four experiments, we postulated a large dwindling and outwashing effect of the injected 200 µM NAD^+^, even when the flow was strongly decreased during application. We concluded that a much higher concentration of NAD^+^ was needed for an effect.

#### 3.3.2. Lung Physiology during EVLP with NAD^+^ 2000 µM Perfusate

**Figure 4:**
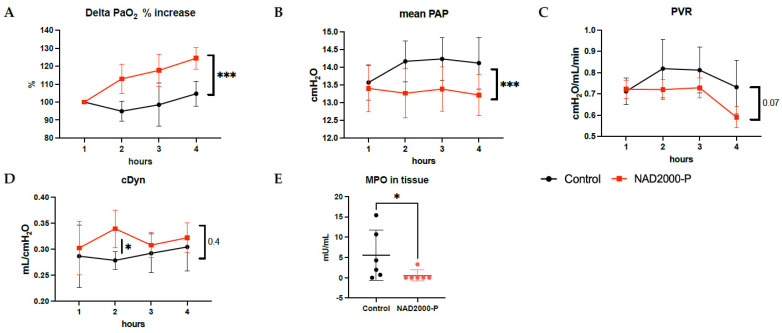
Lung physiology of control or 2000 µM NAD+ in perfusate during 1 to 4 h of EVLP with (**A**) percentage increase in lung oxygen exchange function, (**B**) PAP, (**C**) PVR, (**D**) Cdyn. (**E**) MPO content in tissue after 4 h of EVLP. Note * *p* ≤ 0.05; *** *p* ≤ 0.001. The star is a significant *p* value versus the control (*p* = 0.433, Figure 3E and (**D**)), but with significantly better compliance at 2 h after start of EVLP in the NAD^+^ 2000 µM perfusate (*p* = 0.022, (**D**)). Weight gain of the lungs over time was comparable between the two groups (*p* = 0.262, Figure 3G). The microscopic assessment of lung injuries at the end of the 4 h EVLP period was not significantly different in the NAD^+^ 2000 µM perfusate group (Figure 3H) and in the control (Figure 3K).

Considering the potential dwindling effect during EVLP, we next tested a ten times higher concentration of NAD^+^, 2000 µM, injected bolus in the perfusate of the EVLP system in close proximity to the pulmonary artery every 30 min (at 1 h, 1.5 h, 2 h, 2.5 h, 3 h and 3.5 h of EVLP). This led to favorable and homogenous results for the NAD^+^ 2000 µM perfusate group. The results at 4 h of EVLP are shown in Figure 3 and the hourly detailed changes during EVLP in Figure 4. A significant hourly percentage increase in oxygenation was observed in the NAD^+^ 2000 µM perfusate between the start of application of NAD^+^ at 1 h until 4 h (*p* = 0.0001, Figure 4B), along with a significantly better oxygenation at the end of the experiment (*p* = 0.002, Figure 3A) and oxygenation capacity between 1 and 4 h of EVLP (*p* = 0.002, Figure 3B). The mean PAP was significantly lower at the end of the experiment (*p* = 0.04, Figure 3D) and showed a strong decrease over time with repeated applications of NAD^+^ (*p* = 0.0003, Figure 4B). At the end of the experiment, PVR was also significantly lower (0.017 Figure 3F), and maximal flow capacity significantly higher (*p* = 0.006, Figure 3C) in the NAD^+^ 2000 µM perfusate group. Compliance was overall comparable in the NAD^+^ 2000 µM perfusate group in comparison to the control group.

##### Lung Tissue Biochemical Measurements in NAD^+^ 2000 µM Perfusate

After 4 h of EVLP, we observed significantly lower MPO content in tissue with the NAD^+^ 2000 µM perfusate group, Figure 4E (*p* = 0.026).

##### Perfusate Cytokines and Chemokines in NAD^+^ 2000 µM Perfusate

**Figure 5:**
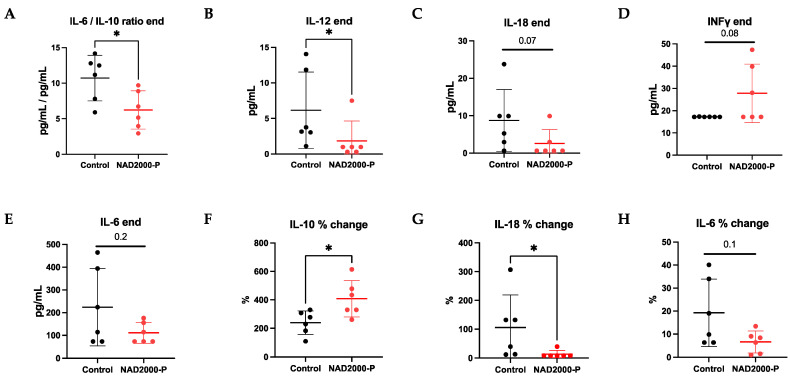
Perfusate cytokines and chemokines of control or 2000 µM NAD+ in perfusate. Data are plotted as effective concentrations (pg/mL) at 4 h of EVLP for (**A**) IL-6/IL-10 end ratio, (**B**) IL-12, (**C**) IL-18, (**D**) IFNγ, (**E**) IL-6, or as percentage concentration changes from 1 h to 4 h EVLP for (**F**) IL-10, (**G**) IL-18, (**H**) IL-6. Note * *p* ≤ 0.05. The stars are significant *p* values versus the control.

At the end of EVLP, we recorded a significantly lower pro-inflammatory IL-6/IL10-ratio (*p* = 0.041), significantly less pro-inflammatory IL-12 (*p* = 0.023), a trend of less pro-inflammatory IL-6 (*p* = 0.193) and IL-18 (*p* = 0.067), as well as a trend of more anti-inflammatory INFy (*p* = 0.078) in the NAD^+^ 2000 µM perfusate group in comparison to the control group (Figure 5, Table 1). Moreover, when the perfusate at 1 h EVLP was compared to 4 h of EVLP, a significant percentage increase in anti-inflammatory IL-10 (*p* = 0.024) and a significant percentage decrease in pro-inflammatory IL-18 (*p* = 0.012) and a trend of decrease in pro-inflammatory IL-6 (*p* = 0.128) was observed by repetitive application of NAD^+^ in the NAD^+^ 2000 µM perfusate group over time. No differences were seen in pro-inflammatory TNFα and anti-inflammatory IL-4 (Table 1). IL-17A in both groups was below the detection limit of the assay.

##### BAL Cytokines and Chemokines in NAD^+^ 2000 µM Perfusate

**Figure 6:**
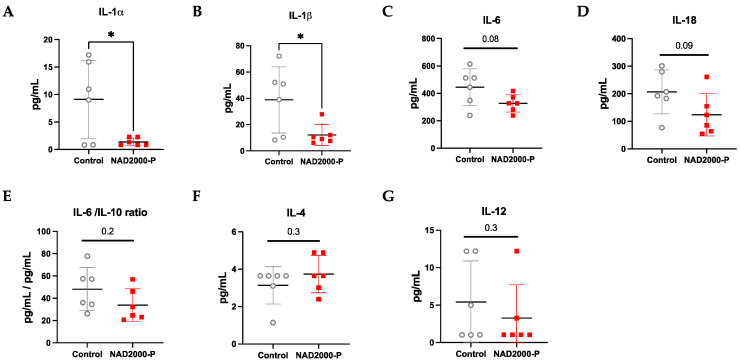
BAL cytokines and chemokines of control or 2000 µM NAD+ in perfusate. Data are plotted as effective concentrations (pg/mL) after 4 h of EVLP for (**A**) IL-1α, (**B**) IL-1β, (**C**) IL-6, (**D**) IL-18, (**E**) IL-6/IL-10 end ratio, (**F**) IL-4, (**G**) IL-12. Note * *p* ≤ 0.05. The stars are significant *p* values versus the control.

In Table 1 and visualized in Figure 6, the BAL collected in the NAD^+^ 2000 µM perfusate group consisted of significantly lower pro-inflammatory IL-1α (*p* = 0.024) and IL-1β (*p* = 0.033) and a non-significant decrease in pro-inflammatory IL-6 (*p* = 0.08) and IL-18 (*p* = 0.093)). The IL-6/IL-10 ratio showed also a trend of reduced inflammation in the NAD^+^ 2000 µM perfusate group (*p* = 0.179). Moreover, there was a slight trend of less pro-inflammatory IL-12 (*p* = 0.251) and anti-inflammatory IL-4 (*p* = 0.317). TNFα was comparable between the groups. IL-2, INFy and IL-17A were below the detection limit of the assay.

##### Biochemical Measurements in NAD^+^ 2000 µM Perfusate

Table 2 shows the dynamics of potassium, calcium, chlorine, lactate, glucose and pH during EVLP for the NAD^+^ 2000 µM perfusate group and control group during EVLP. There was no difference between groups.

## 4. Discussion

Due to chronic organ shortages, extended donor lungs with high allograft ischemic times have steadily increased during the past decades [1]. This increase is still an unresolved problem with high morbidity and mortality due to IRI after lung transplantation. In this EVLP study, we demonstrated that NAD^+^ is a potent additive for the reconditioning and improvement of ischemic rat lungs. Repeated application of NAD^+^ in the perfusate during EVLP improves oxygenation but also reduces PAP, decreases vascular resistance and increases the perfusate flow. Moreover, NAD^+^ reduces both MPO-mediated tissue damage and pro-inflammatory cytokines. In contrast, NAD^+^ applied in the preservation solution did not show favorable effects.

NAD^+^ is required in over 500 enzymatic reactions that play critical roles in the regulation of almost all major biological processes in the human body. Intrinsic NAD^+^ production is governed by at least four known complex pathways capable of regulating NAD^+^ synthesis [19]. We may explain our results as a possible effect of exogenous applied NAD^+^ that increased intracellular and mitochondrial-specific NAD^+^ concentrations [9,19].

### 4.1. NAD^+^ May Reduce Oxidative Stress and Reset Cell Homeostasis

A stable ratio of NAD^+^/NADH is essential to maintain cellular redox homeostasis. The ratio between NAD^+^/NADH is the most crucial factor in different disease models [9,10]. An estimated ratio of 500:1 is considered normal, and any alteration of this relationship can result in cellular damage [18]. During hypoxic stress, an accumulation of mitochondrial NADH and FADH_2_ blocks oxidative phosphorylation via the electron transport chain of glycolysis and the citric acid cycle, consecutively stopping production of the energy carrier adenosine triphosphate (ATP), and finally causes reductive stress and accumulation of ROS [9]. In our model, we significantly injured rat lungs with 14 h of cold ischemic time [21]. In the lungs treated with NAD^+^ during EVLP, we observed a significantly lower level of MPO-mediated tissue damage measured by reduced MPO release. Previous observations suggested that addition of NAD^+^ re-establishes the NAD^+^/NADH ratio and thus reduces ROS and consecutive organ damage [17,24]. Additionally, an NAD^+^ boost has a protective effect due to enhancement of the tissue antioxidant capacity via increased levels of the antioxidant tripeptide glutathione and heightened activities of antioxidant enzymes [24]. Moreover, in the mitochondrial respiration-impaired cells, NAD^+^ improves the lysosomal function and limits the increased proinflammatory profile [25].

### 4.2. NAD^+^ May Directly Act against Hypoxia-Induced Vasoconstriction

A further effect of the repeated application of NAD^+^ may be attributed to the vasodilatative effect of NAD^+^ on the intrinsic mechanism of hypoxia-induced pulmonary vasoconstriction. In pulmonary artery smooth muscle cells, a mitochondrial sensor dynamically detects the response to alveolar hypoxia. Under hypoxic conditions, the NAD^+^/NADH ratio of the cells shifts toward the accumulation of NADH. In brief, this decreases the uncoupled electron transport and results in depolarization, closing of potassium channels and subsequent opening of voltage-gated calcium channels, and increases cytosolic calcium, causing vasoconstriction [11,26]. An NAD^+^/NADH ratio shifted in the direction of NAD^+^ sustains or reestablishes the redox balance and state of vasodilatation. Moreover, the production of the endothelium-derived vasodilator nitric oxide (NO) is catalyzed by nitric oxide synthases that are dependent on NAD^+^ or NADP^+^ as an electron acceptor [12,13]. We indeed observed an improved oxygenation, reduced PAP and increased flow, which may be attributed to vasodilatation. However, one limitation of this work is that we were unable to quantify the impact of NAD^+^ in directly causing vasodilatation. These effects also might have been caused indirectly by reduced tissue damage and a lower inflammation state.

### 4.3. NAD^+^ Reduces the Pro-Inflammatory Milieu

In the lungs treated with NAD^+^ during EVLP, we observed a significantly lower pro-inflammatory milieu. During ischemia, the produced DAMPS activate inflammasomes, which generate caspase-1. Caspase-1 then processes the production of the pro-inflammatory cytokines IL-1β and IL-18, which are both able to activate IL-6 and other pro-inflammatory cytokines [27]. We observed significantly lower levels of IL-18 and a lower IL6/IL10 ratio in the perfusate of lungs treated with NAD^+^ during EVLP. In the BAL of the NAD^+^ treated lungs, we additionally recorded significantly lower levels of IL-1β and not significant but decreased levels of IL-6 and IL-18. Among DAMPS produced during hypoxia, the cytokine IL-1α forms a complex that induces a signaling cascade leading to NF-κB activation and upregulation of multiple pro-inflammatory cytokines [27]. In our study, IL-1α was also significantly reduced in the BAL of lungs exposed to repetitive applications of NAD^+^ during EVLP. Both IL-1α and IL-1β have been shown to be associated with the full spectrum of IRI, from acute rejection to chronic allograft dysfunction [27].

The observed reductions in these pro-inflammatory cytokines by NAD^+^ may be attributed to the combination of different effects: (A) the ability of NAD^+^ to eliminate ROS and consecutively prevent the formation of DAMPs; (B) the capacity of NAD^+^ in efficiently inhibiting inflammasome assembly and consecutive IL-1β release, as shown in two studies with NAD^+^ precursors [28,29]; (C) the effective re-oxygenation of the lung graft due to the vasodilatative effects of NAD^+^, and, as suggested by several studies, (D) a direct alteration of the homeostasis of the innate and adaptive immune system by NAD^+^, finally leading to the observed anti-inflammatory state [10,14,16].

In the lung graft, the most frequent immune cells are alveolar macrophages. The innate macrophage-driven inflammation belongs to one of the first factors inducing the cascade of inflammation during IRI. It was recently shown that a sufficient intrinsic amount of NAD^+^ is essential in maintaining an anti-inflammatory phenotype in resting macrophages [30,31]. Moreover, NAD^+^ induces apoptosis in pro-inflammatory T cells [32,33,34,35], leading to less inflammation potential. When oxidative stress occurs, an imbalanced NAD^+^/NADH ratio leads to the activation of CD4^+^ T cells, to the secretion of pro-inflammatory TNFα, and to a downregulation of immunosuppressive IL-10. Exogenous NAD^+^ reestablishes this imbalance [25]. In line with these findings, we observed a notable increase in protective IL-10 in the perfusate during repeated application of NAD^+^ into the EVLP system over 4 h. An ex vivo study even showed an upregulation of IL-10 secretion by Th1 cells when exposed to extrinsic NAD^+^ without an additional antigen receptor stimulation [14]. In murine models of repeated NAD^+^ administration over days, even a transformation of CD4^+^ T cells and a consecutive robust systemic upregulation of IL-10, IL-4 and TNFy as well as a consecutive strong immunosuppressive effect were observed [14,15,16]. However, it is questionable whether these effects were already present during the few hours of exposure of the lungs to NAD^+^ during our 4 h EVLP experiments, as we detected only a strong trend of higher INFy and a weak trend of higher IL-4 in the perfusate. In our study, we observed no reduction in the pro-inflammatory cytokine TNFα after administration of NAD^+^. However, an increase in TNFα is consistent with two previous studies, which concluded that intracellular NAD^+^ levels upregulate TNFα cytokine production despite final homeostasis results [15,36]. One of the limitations of our EVLP work that were evident from the start were the long-term effects of NAD^+^ on other important immune cells such as B cells, CD8^+^ cells and innate lymphoid cells, which remain to be determined.

### 4.4. No Beneficial Impact of NAD^+^ in the Preservation Solution

To our surprise, we did not observe a beneficial effect of NAD^+^ when two different amounts of NAD^+^ were tested as an additive to the preservation solution during the 14 h of cold ischemic time. Here, the treated lungs showed even a lower oxygenation capacity and a trend toward a lower perfusate flow capacity than the controls, even though there was no apparent edema formation or histopathological lung injury. The chosen amount of NAD^+^ was tested initially in our in vitro bioassay over 24 h as non-toxic for lung cells. However, the mixture of NAD^+^ with the preservation solution and the required buffer for NAD^+^ might have caused toxicity in the tissue. A second reason for this poor and quiet heterogenous result may be attributed in part to the high lability of the NAD^+^ solution, as it rapidly degraded upon heating and was very labile in an alkaline environment, especially in the presence of phosphate, maleate, or carbonate (Sigma-Aldrich product information [37]). Moreover, NAD^+^ is sensitive to light, which may be a reason why it even needed to be injected every 30 min during EVLP for a successful outcome.

### 4.5. Limitations

A stronger impact would have been observed in our EVLP results with a more prolonged period of cold ischemic time for the lungs. Additionally, a further transplantation of the lungs would have given more insight but also additional variables (rat transplantation procedure after EVLP). Moreover, we did not use leucocyte filters during the rat EVLP that were documented to reduce the release of pro-inflammatory cytokine IL-6 in the perfusate and whose absence impaired the quality of the lung grafts [38]. In this study, we did not directly determine the achieved intracellular NAD^+^/NADH ratio necessary for optimal results, which is a limitation of this work. Another limitation was the lack of knowledge of the hypothetical adverse mechanism of NAD^+^ on lungs when given in the preservation solution and during CIT, even though our in vitro bioassay over 24 h was non-toxic for rat lung cells. Another shortcoming of our study was that we only provided data for dosages of 200 or 2000 µM NAD^+^ sequentially injected in the lung during ex vivo perfusion without showing results for more intermediate NAD^+^ dosages. Moreover, we want to emphasize that NAD^+^ dosages used in vivo and in humans are, at 1.3–1.4 mg/kg, much lower than what we tested in our ex vivo perfusion experiments to overcome the observed outwash and dwindling effect.

## 5. Conclusions

Findings from this preliminary study demonstrated that NAD^+^ is a promising agent with both anti-inflammatory properties and the ability to improve lung function after prolonged cold ischemic storage in a rat EVLP model. This observation should be validated in a large animal model, as the rat lung physiology and pathological characteristics presented in this work did not allow a direct clinical translation and should be properly tested before considering NAD^+^ for inclusion in clinical protocols.

## Figures and Tables

**Table 1:** Perfusate and BAL cytokines, chemokines and mediators of wound healing and tissue repair at 4 h EVLP in control and 2000 µM NAD^+^ groups, presented as mean and standard deviation (SD).

	Perfusate		BAL	
	Control	NAD^+^ 2000-P	Control	NAD^+^ 2000-P
	(*n* = 6)	(*n* = 6)	(*n* = 6)	(*n* = 6)
EGF	0.041 (0.029)	0.027 (0.001)	0.612 (0.516)	0.621 (0.415)
Eotaxin	0	0	0	0
Fractalkine	19.03 (9.53)	19.87 (10.00)	225.1 (138.5)	349.2 (235.2)
G-CSF	0	0	0	0
GM-CSF	6.751 (7.84)	14.80 (13.38)	0	0
GRO/KC	3100 (3509)	1780 (1936)	3913 (1599)	2854 (1870)
IFN-γ	17.24 (0.07)	27.80 (13.16)	0	0
IL1-α	4.913 (4.721)	9.237 (10.63)	9.128 (7.104) *	1.377 (0.701) *
IL1-β	9.662 (4.117)	9.062 (5.177)	38.81 (25.18) *	12.17 (7.93) *
IL-2	0	0	0	0
IL-4	3.222 (2.030)	3.430 (1.285)	3.135 (0.997)	3.740 (0.998)
IL-5	7.678 (3.174)	7.582 (3.520)	0	0
IL-6	223.9 (169.9)	110.9 (45.95)	445.4 (133.7)	327.3 (63.0)
IL-10	18.93 (10.43)	18.38 (3.69)	9.962 (3.401)	10.67 (3.249)
IL-12 (p70)	6.160 (5.377) *	1.848 (2.784) *	5.418 (5.481)	3.263 (4.472)
IL-13	2.771 (2.313)	1.708 (1.771)	2.417 (2.468)	1.185 (0.886)
IL-17A	0	0	0	0
IL-18	8.740 (8.247)	2.565 (3.711)	206.8 (79.9)	123.6 (77.0)
IP10	543.8 (62.6)	588.5 (101.2)	783.8 (271.2)	893.8 (277.2)
Leptin	412.8 (106.8)	425.5 (171.8)	2.233 (1.164)	4.426 (4.622)
LIX	35.91 (3.56)	29.84 (7.65)	353.2 (197.7)	309.9 (215.0)
MCP-1	202.3 (86.2)	247.6 (65.3)	174.8 (117.1)	192.8 (123.8)
MIP1-α	482.3 (265.3)	409.0 (165.0)	685.2 (224.0)	552.3 (299.8)
MIP-2	756.5 (843.8)	493.9 (478.5)	5740 (3263)	6107 (3281)
Rantes	44.33 (23.94)	33.24 (8.94)	5.457 (4.735)	4.130 (1.584)
TNF-α	29.73 (19.56)(0.0) *	22.10 (4.79)	18.91 (14.68)	23.40 (19.58)
VEGF	2.850 (3.231)	3.175 (2.229)	554.9 (294.4)	826.4 (361.3)

Cytokines, chemokines and mediators of wound healing and tissue repair (in pg/mL). Note * *p* ≤ 0.05. The stars are significant *p* values versus the control.

**Table 2:** Biochemical measurements in the perfusate of the control and 2000 µM NAD^+^ groups during 4 h of EVLP, presented as mean and standard deviation (SD) over time.

	Control	2000 µM NAD^+^	
	(*n* = 6)	(*n* = 6)	*p*-value
Potassium	3.296 (0.078)	3.217 (0.062)	ns
Calcium	0.677 (0.003)	0.691 (0.003)	ns
Chlorine	92.96 (2.30)	93.75 (1.66)	ns
Lactate	<0.3	<0.3	ns
Glucose	143.7 (8.5)	140.0 (5.7)	ns
pH	7.093 (0.023)	7.069 (0.010)	ns

## Data Availability

The data sets generated during and/or analyzed during the current study are available from the corresponding author on reasonable request.

## Abbreviations

BAL	bronchoalveolar lavage
cDyn	dynamic lung compliance
CIT	cold ischemic time
DAMPS	damage-associated molecular patterns
EVLP	ex vivo lung perfusion
FiO_2_	fraction of inspired oxygen
IRI	ischemia-reperfusion injury
MPO	myeloperoxidase
MTT	Thiazolyl Blue Tetrazolium Bromide Viability Assay
NAD^+^	β-nicotinamide adenine dinucleotide
PAP	pulmonary arterial pressure
PEEP	positive end-expiratory pressure
PVR	pulmonary vascular resistance
ROS	reactive oxygen species
